# Addressing the human resources for health crisis through task-shifting and retention: results from the Africa Health Systems Initiative Support to African Research Partnerships program

**DOI:** 10.1186/1478-4491-12-S1-I2

**Published:** 2014-05-12

**Authors:** Esmé Lanktree, Adrijana Corluka, Marc Cohen, Renée Larocque

**Affiliations:** 1 Global Health Research Initiative, Ottawa, Canada

## Introduction

In 2008, the Global Health Research Initiative (GHRI) invited applications from teams of researchers and decision-makers who were interested in conducting research related to human resources for health and the implementation and use of integrated health information systems in Africa, with special attention to equity considerations. These thematic areas constituted the focus of the Africa Health Systems Initiative - Support to African Research Partnerships (AHSI-RES) program.

The Global Health Research Initiative is a partnership of three Canadian agencies: Foreign Affairs, Trade and Development Canada (DFATD), International Development Research Centre (IDRC), and the Canadian Institutes of Health Research (CIHR). GHRI is hosted at IDRC. AHSI-RES was a five year, $5.9 million CDN research program (2008-2013) supported by Foreign Affairs, Trade and Development Canada ($5 million) and the International Development Research Centre ($900 000). AHSI-RES is the research component of the larger DFATD Africa Health Systems Initiative (AHSI) program. The AHSI program is a 10 year, $450-million CDN commitment (2006-2016) to strengthening national-level health strategies and architecture, and is being implemented by Foreign Affairs, Trade and Development Canada.

The AHSI-RES program’s purpose is to support policy relevant research, knowledge translation and exchange in the program’s thematic areas. The AHSI-RES program emphasized the importance of ongoing interaction, collaboration, and exchange of ideas between researchers and decision-makers to maximize the likelihood that research findings would be used to inform programs and policies. A decision-maker was defined as ‘an individual who makes decisions about, or influences, health policies or practices.’ The program used different approaches in order to build or increase local capacity for research, knowledge translation, and research use. The long-term objective was: “Health systems research allows African decision makers, policy advocates and health service managers to improve health outcomes and reduce disease burden through more efficient and affordable health systems” [1].

Teams were required to include one African researcher and one African decision-maker, both as co-principal applicants. Other African and non-African researchers and decision-makers could be involved as co-applicants or as collaborators. The co-principal applicants had to be affiliated with an institution located in an AHSI-RES geographic area of focus. Geographic areas of focus included: Francophone West Africa (Mali, Burkina Faso, Benin); Great Lakes and Eastern Africa (Tanzania, Uganda, Kenya); and Southern Africa (Malawi, Mozambique, Zambia).

## Selection of research teams

Ten teams were selected from fifty-seven proposals following a rigorous merit review process, which included both researchers and decision-makers. Successful teams had to demonstrate: the ability to engage in interdisciplinary applied research to address complex policy-relevant questions related to the key themes of AHSI-RES; and, the ability to link research, policy, and action to improve health decision-making and programming. Gender, ethics, capacity development, and knowledge translation and exchange were cross-cutting themes featured in AHSI-RES programming.

Selected teams were from seven African countries: Burkina Faso, Mali, Kenya, Tanzania, Uganda, Malawi, and Zambia. Their research focused on two main areas: 1) the recruitment and retention of health workers and the shifting of certain tasks to less specialized health workers (i.e. task-shifting) in response to a severe human resources crisis in the health sector in sub-Saharan Africa; and 2) the role of health information in ensuring greater equity in access to health care. The lessons learned from the uptake and impact of AHSI-RES research for evidence-based practice is presented in a supplement to *BMC Health Services Research*. The AHSI-RES program also produced important results which aim to address the human resources for health crisis through task-shifting, recruitment, and retention. The *Human Resources for Health* journal, due to the relevance of its thematic focus, was selected for the publication of this supplement.

## Focus on human resources for health: task-shifting, recruitment, and retention of health workers

The ten teams supported by this program worked to connect research with policy and action to improve health decision-making and programming in the sub-Saharan region, paying particular attention to the needs of disadvantaged segments of the population. Human resources for health are especially scarce in certain specialties and in certain regions, mainly those which are furthest from urban centres. Understanding how to successfully recruit trained health personnel, maintain their motivation, and retain them in these rural and remote areas has the potential to expand access to health services to the most vulnerable and strengthen health systems in sub-Saharan Africa, a region at the centre of the human resources for health crisis.

The articles in this supplement present research results from Burkina Faso, Tanzania, Uganda, and Zambia.

## Shifting tasks to different cadres: a human resources solution?

A main focus of the project teams is task-shifting, though research on this human resources strategy spans diverse fields including mental health, surgery, community health, and ophthalmology. Primary health care workers provide primary eye care services in Kenya, Tanzania, Malawi, and Madagascar. However, as research described in this supplement shows, their competency scores are low [2], as are the sensitivity and specificity of these signs and symptoms [3], indicating that this task-shifting strategy might need to be refined and/or training strengthened to provide higher quality eye care. The same team recommends that non-physician cataract surgeon training should be supplemented with improvements in equipment, transportation, human resources and organizational support [4], suggesting a health systems approach may be necessary for properly integrating strategies such as task-shifting.

Another team studied job descriptions in Uganda, with the goal of understanding the current scope of the mandates of cadres of health care workers who provide surgical services [5]. These were found not to be explicit regarding surgical tasks, and opportunities were identified to clarify mandates so as to ensure proper training, support, working conditions, and payment.

## Recruitment and retention

Recruitment, motivation, and retention have also been major foci of the AHSI-RES program research teams. Although different recruitment and retention strategies have been put in place by national governments, the effectiveness of these strategies is not often evaluated. One team chose to explore this in Zambia. Despite studying nineteen human resource strategies currently being implemented, the research did not identify a strong association between any of these and job satisfaction or likelihood of leaving their job [6]. In fact, the health workers’ characteristics and the conditions in which they find themselves may have more weight. The government of Zambia is currently in the process of refining their human resources for health strategies.

Researchers also studied Burkina Faso’s regionalized health personnel recruitment policy and questioned the sustainability of the policy given the absence of incentives [7]. The researchers also explored which incentive packages would be best at retaining health workers in rural areas [8]. As with the Zambia team, health workers’ characteristics were identified as having an influence on retention, although specific items included in the incentive packages were preferred (e.g. provision of housing).

## Next steps

The task-shifting and retention and recruitment research conducted within the context of the AHSI-RES program has uncovered important areas of focus for refining current human resources for health strategies, and approaches to evaluate whether these are producing the intended results. They also raised important issues to consider. Retention and recruitment strategies may not have an effect if they are designed without considering the needs or preferences of health workers. Likewise, task-shifting is not necessarily a solution in itself, but must be complemented by proper training, support, supervision, monitoring and evaluation, and a clear understanding of scope of practice. This indicates a need for clear guidelines, regulation, and recognition of the work of task-shifted health workers.

## Competing interests

Adrijana Corluka and Renée Larocque serve as Senior Program Officers, Marc Cohen serves as Program Officer, and Esmé Lanktree serves as Program Management Officer, with the Global Health Research Initiative. The Global Health Research Initiative supported the assembly and publication of this supplement. The views expressed in this introductory article are those of the authors alone and do not represent the views of the Global Health Research Initiative, the International Development Research Centre, the Canadian Institutes of Health Research, nor Foreign Affairs, Trade and Development Canada.

## Declarations

The publication costs associated with this article are funded by Foreign Affairs, Trade and Development Canada and the International Development Research Centre through the Global Health Research Initiative.

This article has been published as part of *Human Resources for Health* Volume 12 Supplement 1, 2014: Addressing the human resources for health crisis through task-shifting and retention: results from the Africa Health Systems Initiative's research component. The full contents of the supplement are available online at http://www.human-resources-health.com/supplements/12/S1
